# Understanding the functional inflammatory factors involved in therapeutic response to immune checkpoint inhibitors for pan-cancer

**DOI:** 10.3389/fphar.2022.990445

**Published:** 2022-09-01

**Authors:** Yanmeizhi Wu, Shan Yu, Hong Qiao

**Affiliations:** ^1^ Department of Endocrinology, The Second Affiliated Hospital of Harbin Medical University, Harbin, China; ^2^ Department of Pathology, The Second Affiliated Hospital of Harbin Medical University, Harbin, China

**Keywords:** immune checkpoint inhibitors, inflammatory factors, programmed death-1, immune-related adverse events, tumor microenvironment

## Abstract

Immune checkpoint inhibitors (ICIs) fight tumor progression by activating immune conditions. The inflammatory factors are playing a functional role in programmed death-1 (PD-1) or other immune checkpoints. They are involved in regulating the expression of programmed death ligand-1 (PD-L1), the only predictor recognized by the guidelines in response to ICIs. In addition, abundant components of the tumor microenvironment (TME) all interact with various immune factors contributing to the response to ICIs, including infiltration of various immune cells, extracellular matrix, and fibroblasts. Notably, the occurrence of immune-related adverse events (irAEs) in patients receiving ICIs is increasingly observed in sundry organs. IrAEs are often regarded as an inflammatory factor-mediated positive feedback loop associated with better response to ICIs. It deserves attention because inflammatory factors were observed to be different when targeting different immune checkpoints or in the presence of different irAEs. In the present review, we address the research progresses on regulating inflammatory factors for an intentional controlling anti-cancer response with immune checkpoint inhibitors.

## 1 Introduction

Immunotherapy modulates the body’s immune system to fight immune evasion and immune silencing of tumors. Immune checkpoint inhibitors (ICIs) have the effect of stimulating activation and proliferation on T cells, so it is more beneficial to destroy tumor cells. Currently available immune checkpoint inhibitors are mainly programmed death-1 (PD-1) and its ligand (PD-L1), targeting cytotoxic T lymphocyte antigen-4 (CTLA-4). Further ICIs are being discovered and studied (Parry et al., 2005; Rosenberg et al., 2016), such as Lymphocyte Activation Gene-3 and T cell immunoglobulin mucin-3. Responses to ICIs have only been observed in certain solid tumors, such as gastrointestinal malignancies and unresectable or metastatic melanoma. And it is often accompanied by the possibility of resistance to ICIs (Haslam and Prasad, 2019; Puccini et al., 2020). The major reasons that account for diverse responses to ICIs are the contributions of multiple components of the tumor microenvironment (TME), including immune cells, fibroblasts, and the extracellular matrix (ECM). Thus, the interaction of inflammatory factors with components in the TME modulates responses to ICIs from different perspectives.

The number of patients being treated with ICIs is increasing, and thus cases of immune-related adverse events (irAEs) are also reported in a wide range of organs (June and Bluestone, 2017; Poto et al., 2022). Although the presence of irAEs is generally believed to be associated with better responses to ICIs, unpredictable and uncontrollable irAEs are a major obstacle preventing a complete course of ICIs therapy. The types of inflammatory factors involved in different irAEs are different, and the inflammatory factors associated with different therapeutic targets are not the same (Teulings et al., 2015; Berner et al., 2019). In addition, the discordance of inflammatory factors in TME and irAEs has also led to a new hypothesis that inflammatory factors mediate different signaling pathways involved in tumor response to ICIs and triggering of tissue-specific irAEs.

We aim to highlight the role of inflammatory factors in the TME on the response to ICIs, and also to address the involvement of inflammatory factors in the occurrence of irAEs.

## 2 Inflammatory factors on response to immune checkpoint inhibitors in the tumor microenvironment

**FIGURE 1:**
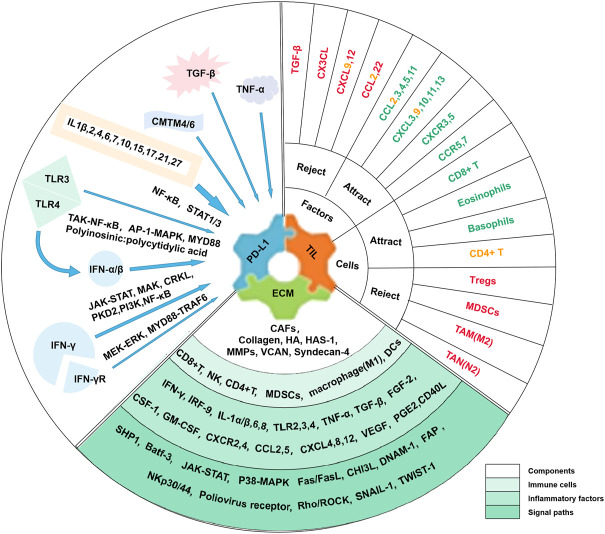
Influence of Inflammatory Factors on Responses to ICIs in TME. In the TME, inflammatory factors affecting tumor response to ICIs were mainly associated with three factors, including PD-L1 abundance, TIL density, and ECM status. Many inflammatory factors affect PD-L1 expression, such as IFN-γ. Inflammatory factors and immune cells are both involved in influencing TIL infiltration with different roles. Among them, green factors or cells attract T cells to homing or promote TIL infiltration, while red ones reject T cell homing or inhibit TIL infiltration. Factors or cells that are yellow have been reported to have a dual effect. Factors affecting ECM status include biological and physical factors, and the components, immune cells and inflammatory factors, and signal paths involved are listed.

Research on immune checkpoints has shown that most of them act as “brakes” in immune function, i.e., ICIs reactivate T cells to more effectively clear cancer cells (Ljunggren et al., 2018). At present, ICIs widely used in clinical applications mainly include anti-PD-1/PD-L1 and anti-CTLA-4. Additional immune checkpoint molecules are under study (Bagchi et al., 2021). The combination of PD-L1 and PD-1 will accelerate the apoptosis of immune cells, especially those with PD-1 positive. CTLA-4 also inhibits T cell activity and is up-regulated upon T cell activation (Valk et al., 2008). However, PD-L1 was a predictor for the response to ICIs rather than CTLA-4. One classification of TME is based on tumor-infiltrating lymphocytes (TILs) and PD-L1 expression, which are the main predictors of response to ICIs (Powles et al., 2014). Among them, one type with PD-L1 positive and abundant TIL infiltration had the best response to ICIs treatment (Teng et al., 2015). Furthermore, cancer ECM influences the response to immunotherapy through secreted regulators and suppressors (Umair et al., 2018; Bagaev et al., 2021). The ECM status provides important complementary information on the capture capacity of T cells (Mariathasan et al., 2018) and is considered a major common denominator of anti-ICIs (Jensen et al., 2021). Among them, cancer-associated fibroblasts (CAFs) are involved in constituting ECM fibrosis to hinder T cell infiltration and T cell activity, which produces a marked effect on immunotherapy resistance (Wang et al., 2018a; Mariathasan et al., 2018). The following summarizes the effects of inflammatory factors on the response of ICIs in TME from three aspects: PD-L1 abundance, TIL density, and ECM status (Figure 1).

### 2.1 Inflammatory factors regulating programmed death ligand-1 abundance

The abundance of PD-L1 in TME could predict therapeutic response to ICIs in multiple cancers, such as melanoma (Caroline et al., 2015) and cancer in lung (Borghaei et al., 2015) and bladder (Rosenberg et al., 2016). Inflammatory-related signaling was confirmed to regulate the expression of PD-L1 (Ji et al., 2015) but was detected not only on T cells (Fehrenbacher et al., 2016). PD-L1 is commonly detected by adaptive and innate immune cells, as well as epithelial cells, especially under inflammatory conditions (Sharpe et al., 2007). Many classical inflammatory factors have a hand in the regulation of PD-L1, which are discussed below respectively.

#### 2.1.1 Interferons

Interferons (IFNs) are secreted in large quantities by various activated immune cells, mainly by T cells and natural killer cells (NK cells). Importantly, PD-L1 of target cells can be triggered by IFN-γ (Garcia-Diaz et al., 2017). T cells secreted IFN-γ through the JAK1/JAK2-STAT1/STAT3 pathway (Karachaliou et al., 2018; Na et al., 2018; Thiem et al., 2019; Fujita, 2021). The JAK1/JAK2-STAT1 pathway has also been observed to stimulate IFN-γ secretion in NK cells (Bellucci et al., 2015). Further, IFN-γ receptor can also stimulate JAK-STAT, and STAT1 signaling is preferred (Platanias, 2005). In contrast, IFN-γ induces protein kinase D isoform 2 (PKD2), an important isoform that inhibits PD-L1 and promotes a strong antitumor immune response (Chen et al., 2012). Silencing of Male Germ Cell Associated Kinase (MAK), CRK Like Proto-Oncogene (CRKL) and PI3K signaling pathways also impair PD-L1 depending on IFN-γ (Garcia-Diaz et al., 2017). In melanoma, nuclear factor-κB(NF-κB) signal is required for IFN-γ to upregulate PD-L1 (Gowrishankar et al., 2015). Furthermore, IFN-γ receptor 1 inhibition can reduce PD-L1 expression in acute myeloid leukemia mice through the MEK-ERK and myeloid differentiation factor88 (MYD88)-tumor necrosis factor receptor-associated factor6 (TRAF6) pathways (Abiko et al., 2015). Since IFN-γ of TME is generally considered to be beneficial for immunotherapy, tumor cells may undergo survival-stressed proliferation when the IFN-γ signaling pathway is defective (Abril-Rodriguez and Ribas, 2017). This is one of the currently recognized causes of adaptive resistance during immunotherapy (Yi et al., 2018). Thus, an intact IFN-γ signaling pathway is a determinant for robust antitumor effects that persist throughout the course of ICIs treatment. It is worth noting that the close relationship between IFN-γ levels and PD-L1 was not necessarily observed in all cell types. For example, when sarcoma mice were treated with an IFN-γ blocking antibody, PD-L1 was largely abolished on tumor cells but not on tumor-associated macrophages (Noguchi et al., 2017). Therefore, more studies on the interaction of IFN-γ and PD-L1 need to be carried out.

IFN-α/β, also induces PD-L1 in cancer cells, endothelial cells and leukocytes (Schreiner et al., 2004; Eppihimer et al., 2015; Garcia-Diaz et al., 2017). IFN-α sensitizes the STAT1 signal, further increasing PD-L1 (Meng et al., 2020). However, there are limited reports on the correlation of IFN-α/β with response to ICIs, which may be due to the small effect of IFN-α/β on PD-L1 directly.

#### 2.1.2 Toll-like receptors

Toll-like receptors (TLRs) family, secreted by both myeloid cells and lymphocytes (Loke and Allison, 2003; Gang et al., 2013), connect innate immunity and adaptive immunity. On the one hand, PD-L1 expression is regulated by TLR4 through MyD88-dependent or MyD88-independent pathways (Lu et al., 2008). Besides, TLR4 activates the downstream NF-kB signaling pathway through transforming growth factor kinase 1 (TAK1) and activates the MAPK pathway through the transcription factor AP-1 (Qian et al., 2008). On the other hand, IFN-α production is activated by TLR4 ligands in plasmacytoid dendritic cells (PDCs) (Zhang et al., 2020). TLR3 can also cause increased PD-L1 expression in DCs (Pulko et al., 2009), endothelial cells (Cole et al., 2011) and neuroblastoma cells (Boes and Meyer-Wentrup, 2015) under conditions stimulated by polyinosinic:polycytidylic acid. However, the relevant mechanism is still unclear.

#### 2.1.3 Interleukin (ILs)

Interleukin (ILs) are a class of lymphokine that interacts between immune cells. On the one hand, a number of ILs upregulate PD-L1 on immune cells, including IL-10 (Cole et al., 2011) and IL-17 (Zhao et al., 2011) in monocytes, IL-4 (Ou et al., 2012) and IL-6 (Kim et al., 2008) in dendritic cells. IL-1β upregulates PD-L1 *via* the NF-κB (Kondo et al., 2010), and IL-27 *via* STAT1 and STAT3 signaling pathways (Karakhanova et al., 2011). In addition, IL-2, 7, 15 and 21 also increased PD-L1 *in vitro* (Kinter et al., 2008). On the other hand, IL in tumor cells can also cause high PD-L1, like IL-4, IL-17 in prostate and colon cancer cells (Wang et al., 2017), IL-27 in ovarian cancer cells (Carbotti et al., 2015), IL-10 in myelodysplastic syndrome blasts (Kondo et al., 2010), renal cell carcinoma (Quandt et al., 2014). IL-12-mediated regulation of PD-L1 may be more complex because IL-12 raises PD-L1 *via* NF-κB in macrophages while lowering PD-L1 when IFN-γ lacking (Xiong et al., 2014).

#### 2.1.4 Other factors

Transforming growth factor (TGF)-β production by CD8+ T cells was required for cells to consistently express PD-L1 (Baas et al., 2016). Meanwhile, TGF-β induced DCs to express PD-L1 *in vitro* in lung cancer (Ni et al., 2012) and hepatocellular carcinoma (Song et al., 2014), respectively. Tumor necrosis factor (TNF)-α in endothelial cells was observed to be relevant to high PD-L1 (Mazanet and Hughes, 2002). More reports are in autoimmune diseases such as Paget’s disease (Iga et al., 2019) and rheumatoid arthritis (Wasén et al., 2018). The regulation of PD-L1 expression by these promising inflammatory factors in the TME and further clarification of their roles in response to ICI therapy are warranted. In addition, CKLF (Chemokine-like factor)-like MARVEL transmembrane domain containing (CMTM)4/6 present on the cell surface binds to PD-L1 on protein level rather than transcription (Mezzadra et al., 2017).

### 2.2 Inflammatory factors regulating tumor-infiltrating lymphocyte density

Along with PD-L1 positivity, tumors respond well to ICIs when TILs are abundant and show drug resistance otherwise (Na et al., 2018). Thus, TIL density acts as a predictor of response to treatment with ICIs, too. It has been found that abundant TILs are associated with both adaptive upregulation of PD-L1 and the clinical benefit of immunotherapy (Xing et al., 2018). Pre-existing TILs are liberated by PD-L1/PD-L1 inhibitors and then promote tumor regression (Yagi et al., 2017; Tomioka et al., 2018). However, TILs on response to anti-CTLA-4 therapy is controversial. Baseline TIL status before treatment has been reported to predict a good therapeutic effect in melanoma (Chen et al., 2016), but no prognostic benefit has also been observed (Huang et al., 2011). Although anti-CTLA-4 is not prominent in immunotherapy as a monotherapy, it is being clinically tested in a variety of cancers as an effective means of enhancing anti-tumor response when applied with other therapies, such as chemotherapy and radiotherapy. This section focuses on those inflammatory factors and immune cells in the TME that attract or reject homing T cells and thus positively or negatively correlate with ICI responses.

#### 2.2.1 Tumor-infiltrating lymphocyte cells associated with immune checkpoint inhibitor responsiveness

TCRs (T cell receptors) are massively amplified in metastatic pancreatic cancer following anti-CTLA-4 (Hopkins et al., 2018). Within the TME, the enrichment of T cells with the same TCR is a hallmark of effective treatment with ICI therapy (Ro et al., 2017; Arakawa et al., 2019). CD8+ T cells are regarded as the key to “stem cell-like” antitumor immunity due to their unique expression of the transcription factor TCF1 (Siddiqui et al., 2019). After ipilimumab, those patients with CD45RA- had better responses, both for CD4+ T and CD8+ T cells (Subrahmanyam et al., 2018). In a study of nivolumab in non-small cell lung cancer, 18% of patients (23/126) exhibited complete or partial responses after treatment. Responders had more CD62LlowCD4+ T cells in the predose circulation than non-responders (Kagamu et al., 2020). Similar results in metastatic melanoma had shown that 9 of the 32 patients responded to ICIs. More memory CD4+ T cells were in the cancer tissues of the 9 responders, which were not only related to the expression of CD62L, but also to CCR7 and CD28 (Sade-Feldman et al., 2019). Therefore, the frequency of pre-treatment TIL populations and their dynamic changes may serve as important predictive biomarkers for distinguishing between ICI responders and non-ICI responders.

#### 2.2.2 Inflammation factors related to T cell homing

**FIGURE 2:**
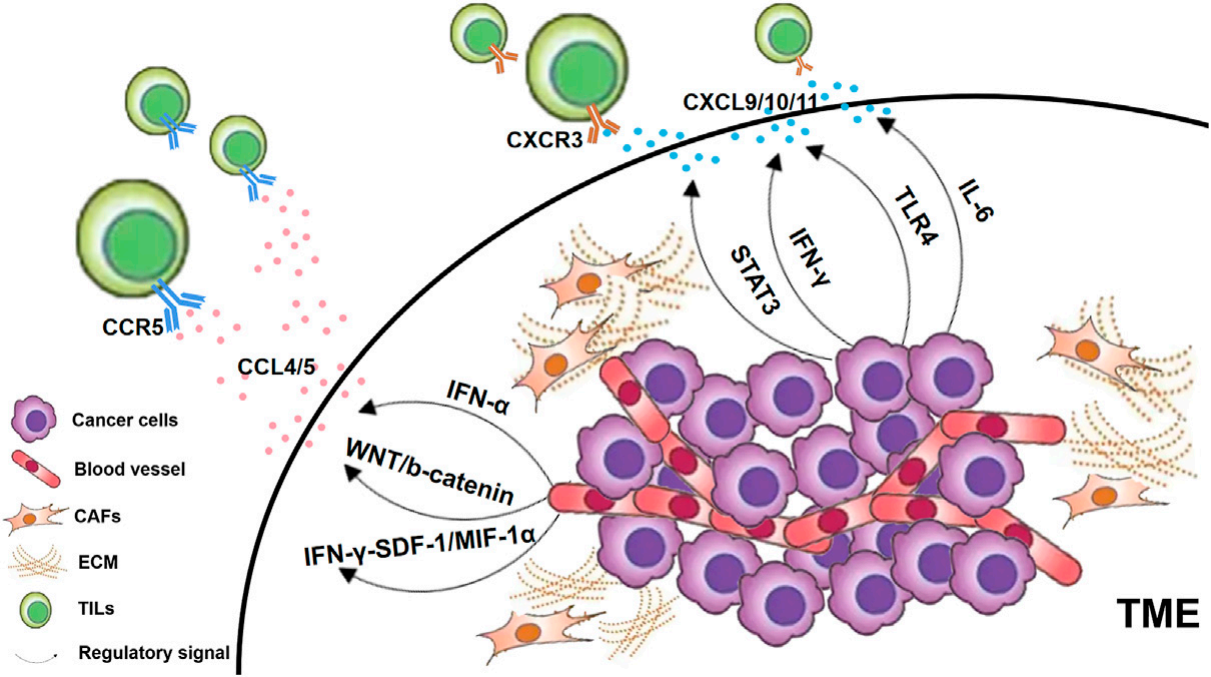
Chemokines regulated by inflammatory factors. In general, cancer cells in the TME, CAFs and ECM can all secrete chemokine ligands. Those T lymphocytes that express chemokine receptors on their cell surfaces are attracted to specific chemokine ligands and thus home to the TME as TILs. CXCR3-CXCL9/10/11 is a key chemokine reported to be associated with TILs infiltration, which is regulated by various signaling pathways as shown in the figure. In addition, CCR5-CCL4/5 has also been found to be associated with the infiltration of TILs through multiple pathways.

Chemotactic cytokines are a class of small cytokines that induce the orientation of responding cells into the site of infection during the immune response. Some chemokines are themselves pro-inflammatory factors, such as IL-8 (Maekawa et al., 2022), while others are regulated by inflammatory factors (Figure 2). Chemokine CC receptor (CCR) 7 binds to its selectin ligand, and chemotactic mature T cells migrate to specific targets. The phenomenon of immune desertification in the TME is a consequence of CCR7 deficiency (Berghuis et al., 2011). The expression of chemokine CC ligand (CCL) 5 is guided by IFN-α and gathers T cells in prostate cancer (Harlin et al., 2009). When triggering WNT/b-catenin, CCL4 downregulation results in failure of effector T cell recruitment and activation (Stefani et al., 2015). Both TLR4 and IL-6 levels were positively correlated with chemokine CXC receptor (CXCR) 3 expression, which may regulate the infiltration of CD+8 T cells (Muthuswamy et al., 2016). In tumor-infiltrating PDCs, CXCR4 is regulated to activate the TNF-α through NF-κB (Han et al., 2021). Inhibition of IFN-γ-induced SDF-1/MIP-1α pathway also reduces CXCR4 and CCR5 (Creery et al., 2004). The chemokine CXC ligand (CXCL) 9 and CXCL10 also contributed to the infiltration of TILs (Liu et al., 2015; Peng et al., 2015). STAT3 signaling can downregulate the CXCR3/CXCL10 axis in CD8+ T cells (Yue et al., 2015), thereby reducing their ability to penetrate the TME. CXCL9 and CXCL10 can be activated *via* IFN-α to promote TILs increasing in the cancer of the prostate gland (Harlin et al., 2009). CXCL11 was correlated with TLR4 levels positively (Muthuswamy et al., 2016). Regardless of tumor subtype, T cells from breast cancer patients treated with anti-PD-1 were clonally expanded, mediated CD8+ T cell homing *via* CXCL13, and attracted CD4+ T cells by CXCR5 (Bassez et al., 2021). In addition, CCR5, CCL2, CCL3, CCL11 and CX3CL1 were also positively associated with T cell infiltration (Peng et al., 2015; Manou et al., 2020; Bassez et al., 2021).

However, chemokines do not always work as expected (Bassez et al., 2021). A study of patients with metastatic urothelial carcinoma who received PD-L1 blocker found that TILs could not reach the tumor center and were trapped in the surrounding matrix (Mariathasan et al., 2018). Tumor-derived chemokines are one of the main reasons for misdirecting activated T cells to the stromal cells surrounding the tumor. For example, stromal cells surrounding pancreatic tumors produce CXCL12, which attracts effector T cells and prevents them from entering the tumor core (van der Woude et al., 2017). The CXCL9/CXCR3 axis may also have a dual role in promoting T cell migration and tumor cell metastasis (Billottet et al., 2013). High expression of CCL2, CCL22, and CXCL12 normally attracts immunosuppressive cells (Harlin et al., 2009). For non-small cell lung cancer, CCL2 blockade reduces immunosuppression and enhances cancer immunotherapy (Fridlender et al., 2010).

The TGF-β has been concerned due to its negative impact on T cells. Blocking the TGF-β signaling pathway contributes to the transition of the TME to an immune-inflammation-rich state, which has significant advantages in tumor control when combined with ICI (Mariathasan et al., 2018; Ravi et al., 2018). Moreover, stimulation of PD-1/PD-L1 by TGF-β receptor (TGF-βRII) results in a reduction in the host’s rejection of the graft, suggesting that similar processing may also be involved in the TME (Baas et al., 2016).

#### 2.2.3 Regulatory immune cells

Several regulatory immune cells influence TIL infiltration (Gabrilovich, 2017; Mantovani et al., 2017) (Table 1), mainly including regulatory T (Treg) cells, myeloid-derived suppressor cells (MDSCs) and monocytes.

**TABLE 1:** Regulation of TIL infiltration by Regulatory immune cells.

		Inflammatory factors	Outcomes	References
APC	Treg	Depletion of IL-2	Inhibiting the proliferation of effector T cell	Setoguchi et al. (2005)
		Expression of LAG-3	Inhibiting the activation of T cell	Huang et al. (2004)
		TGF-β, IL-10	Killing effector T cells	Takahashi et al. (1998), Camisaschi et al. (2010)
		IDO		Mellor and Munn (2004)
		Perforin, granzyme		Mantovani et al. (2017)
	MDSCs	STAT3	Promoting the expression of PD-L1	Ko et al. (2009)
		NADPH	Inhibiting the migration of T cell	Nagaraj et al. (2007)
		COX-2/PGE-2	Inhibiting T cell reactivity	Obermajer et al. (2011)
		Arginase, iNOs		Orillion et al. (2017), ChristmasEntinostat et al. (2018)
		CXCR2, CSF-1R/CSF-1		Mok et al. (2014), Kumar et al. (2017a)
		5-aza-2′-deoxycytidine		Daurkin et al. (2010)
	TAMs	LILRB2-SHP1/2-ERK/P38	Differentiate to TAM-1	Gordon et al. (2017)
		CD47	Promoting TAM activity	Li et al. (2019)
	TANs	CCL5, CXCL9, CXCL10	Eosinophils promote TAM-1 differentiation and T cell migration	Eruslanov et al. (2014)
		CCL3, CCL4	Basophils promote T cell infiltration	Singhal et al. (2016)
	Monocytes	—	Contributes to anti-PD-1/CTLA-4 responses	Carretero et al. (2015); Sektioglu et al. (2016)
	DCs	EZH2	Contributes to anti-PD-1 responses	Bolton et al. (2015)

Tregs can either restrict the amount of effector T cells by depleting IL-2 (Setoguchi et al., 2005) or inhibit the function of effector T cells by producing TGF-β and IL-10 (Takahashi et al., 1998; Camisaschi et al., 2010). In turn, TGF-β and IL-10 expand Treg cells (Chen et al., 1994). In addition, Treg cells produce perforin and granzymes to kill effector T cells (Mantovani et al., 2017). Activated Treg cells express LAG-3 (Huang et al., 2004) and CTLA-4, which promote indoleamine 2,3-dioxygenase (IDO) secretion (Mellor and Munn, 2004), thereby causing T cell dysfunction (Uyttenhove et al., 2003). Small numbers of Tregs are associated with increased ICIs response and patient survival (Read et al., 2006; Friedline et al., 2009). In bladder cancer patients, however, CTLA-4 blockade resulted in an increased proportion of Tregs (Liakou et al., 2008). This suggests that the baseline status of immune cells and ICIs-related changes are of different predictive significance and that effector T cells/Treg ratio may be a more effective method for predicting response to treatment with ICIs (Sharma et al., 2021). Significantly, the effect of anti-CTLA-4 depends on antibody-dependent cytotoxic activity (ADCC) to deplete CTLA-4-expressing Treg cells in the TME, and its deletion will abolish the anti-tumor effect of anti-CTLA-4 (Bulliard et al., 2013; Selby et al., 2013). PD-1 inhibitors on Treg cells remain unclear.

Correlations between MDSCs abundance and response to ICIs have been revealed in numerous clinical trials (Meyer et al., 2014; Weber et al., 2016). MDSCs suppress the immune system by expressing PD-L1 (Noman et al., 2014), secreting IL-10 and TGFβ (Hart et al., 2011; Trikha and Carson3rd, 2014), and recruiting Tregs with CD40 (Pan et al., 2010). MDSCs impede response to ICIs not only with ipilimumab (Liakou et al., 2008) but also with anti-PD-L1 therapy (McDermott et al., 2018). MDSCs can reduce T cell reactivity (Messmer et al., 2015). In renal cell cancer, the inhibition of STAT3 aggregates MDSC, further leading to T cell suppression (Ko et al., 2009). MDSCs abolish the directional migration of CD8+ T cells when upregulated by NADPH oxidase (Nagaraj et al., 2007; Lu et al., 2011). Decreased recruitment and differentiation of MDSCs through obstruction of COX-2/PGE2 resulted in improved CTL frequency and immune responses (Obermajer et al., 2011). In addition, the immunosuppressive function of MDSCs was suppressed by downregulating arginase and iNOS, resulting in a shift in tumor dynamics towards more responsive ICIs (Orillion et al., 2017; ChristmasEntinostat et al., 2018). Targeting CXCR2 reduces the number of MDSCs in pancreatic cancer (Steele et al., 2016). Treatment against the receptors of MDSCs or their ligands CSF-1R/CSF-1 combined with immunotherapy can improve antitumor T cell activity and tumor regression (Mok et al., 2014). The combined use of CSF-1R inhibition and CXCR2 antagonism can also reduce the number of MDSCs and improve anti-PD-1 efficacy (Kumar et al., 2017a). Targeted drugs that inhibit the recruitment of tumor-infiltrating CXCR2+ MDSCs can enhance the response of ICIs (Sun et al., 2019). In addition, inhibition of MDSCs by the DNA demethylating agent 5-aza-2′-deoxycytidine also promotes antitumor immune responses (Daurkin et al., 2010).

Increased frequency of circulating monocytes at baseline predicts better ICI response (Martens et al., 2016; Krieg et al., 2018). Macrophages, granulocytes and DCs are of importance. The phagocytic capacity of tumor-associated macrophages (TAMs) is regulated by the PD-1/PD-L1 (Gordon et al., 2017). Differentiation of PD-1+ TAMs impairs effector T cell function (Li et al., 2019). Type 1 polarization (TAM-1) of TAMs is prevented by blocking leukocyte immunoglobulin-like receptor B2 (LILRB2), which improves anti-PD-L1 responses (Chen et al., 2018). CD47 blockade increases the efficiency of anti-PD-L1 in melanoma to by modulating the activity of TAMs (Sockolosky et al., 2016). Tumor-associated neutrophils (TANs) can be divided into type 1 (TAN-1) and type 2 (TAN-2), the former stimulating infiltration of effector T cells (Eruslanov et al., 2014) and the latter an immunosuppressive phenotype (Singhal et al., 2016). Increased eosinophils contribute to the polarization of macrophages in favor of TAM-1, which is further mediated by CCL5, CXCL9 and CXCL10 (Carretero et al., 2015). Conversely, basophils attract CD8+ T cells in the TME by producing CCL3 and CCL4 (Sektioglu et al., 2016). Differentiation of peripheral DCs also modulates the response to anti-PD-1 (Bolton et al., 2015).

### 2.3 Inflammatory factors participating in extracellular matrix

Cancer ECM is formed through secreted regulators and repressors (Ho et al., 2020). CAFs participate in the process of tumor fibrosis and contribute to the ECM (Jensen et al., 2021). Fibrosis of the ECM through TGF-β signal (Nissen et al., 2019), is considered to be one of the key factors affecting the response to immunotherapy (Bagaev et al., 2021). A combined anti-CTLA-4 and anti-PD1 approach was linked to ECM components to enhance retention within the ECM, suggesting that ECM modulation may be an effective approach to enhance the efficacy of ICIs therapy (Ishihara et al., 2017).

#### 2.3.1 Extracellular matrix formation affects immune cells and inflammatory factors

The formation of ECM determines the migration and localization of immune cells to a certain extent (Hynes, 2009; Hallmann et al., 2015). Based on TME extracellular components, tumors are classified as immune-inflammatory tumors, immune-rejective tumors, and immune-desert tumors (Hegde and Chen, 2020). Immunorejecting tumors respond worse to ICIs than immunoinflammatory tumors, as T cells are blocked away from the tumor (Tumeh et al., 2014; Chen and Mellman, 2017). First, the dense ECM acts as a physical barrier to hinder the infiltration of TILs. Abundant collagen blocks homing T cells despite being activated (Evanko et al., 2012). Elevated collagen level causes patients to resist anti-PD-1/PD-L1 (Peng et al., 2020). Hyaluronic acid (HA) also prevents the move of effector cells (Jacobetz et al., 2013). Chemokine-dependent T cells were observed to infiltrate restricted due to the dense HA surrounding lung cancer tissue (Salmon et al., 2012). Second, immune cells are regulated by the composition of the ECM (Cooper et al., 2016). Collagen inhibits T cell activity by signaling through SHP-1 (Peng et al., 2020). HA promotes inflammation in the TME and increases Treg activity through TLR signaling (Nikitovic et al., 2015). Matrix metalloproteinases (MMPs) promote tolerance polarization in DCs by binding to TLR2 (Godefroy et al., 2014). Vascular endothelial growth factor (VEGF), which is abundantly present, also has inhibitory effects on DCs (Burbage and Amigorena, 2020). Chondroitin sulfate proteoglycan (VCAN) is directly associated with the reduction of TIL (Gorter et al., 2010), as it prevents T cell adhesion and migration (Peng et al., 2020). Meanwhile, it can also indirectly inhibit CTL infiltration by recruiting MDSCs (Wight et al., 2014) and regulating Batf3 DCs (Hope et al., 2017). Third, some ECM components influence inflammatory factor levels. For example, MMP-9 increases TNF-α, IL-1β, IL-6 levels (Liao et al., 2021). Low doses of collagen synergize with CXCL12 to induce the release of CD40L *via* p38-MAPK activation (Nakashima et al., 2020). Collagen peptides can significantly inhibit the secretion of IL-1β, TNF-α and PGE2 (Wang et al., 2018b). Thus, type III collagen propeptide is a predictor of metastatic melanoma response to ICIs (Hurkmans et al., 2020), and type IV collagen fragments can also identify melanoma patients who benefit from anti-CTLA-4 (Jensen et al., 2020).

#### 2.3.2 Immune cells and inflammatory factors affect extracellular matrix remodeling

Remodeling of the ECM is thought to be a key factor in immune cell trafficking and activation of the immune cycle (Huse, 2017). In bladder cancer patients, PD-L1 expression is discordant at metastatic sites and the primary tumor, suggesting the dynamic nature of the ECM (Mukherji et al., 2016). TGF-β has been reported to increase the synthesis and deposition of type I collagen and downregulate MMP-2 (Türlü et al., 2021). Activation of the RhoA/ROCK signaling pathway and transcription of SNAIL1 and TWIST1 genes are both triggered by TGF-β (Gaggioli et al., 2007; García-Palmero et al., 2016; Chung et al., 2021). At the same time, TGF-βR is also necessary for the deposition of collagen and fibronectin (Shuang and Chakrabarty, 2010; Robert et al., 2018). TNF-α and fibroblast growth factor (FGF)-2 are also important adjuvants in the process of promoting collagen synthesis (Katsumata et al., 2019; Muchová et al., 2021). In contrast, IL-1α/β promotes IL-6 and IL-8 and inhibits ECM, by regulating the expression of Versioncan (Chang et al., 2017; Osei et al., 2020). Interferon regulatory factor 9 regulates VCAN transcription independently of JAK-STAT signaling (Brunn et al., 2021). Immune cells are also involved in engineering ECM components (Bhattacharjee et al., 2019). For example, macrophages produce Hyaluronic Acid Synthase 1 *via* TLR2 and Syndecan-4 *via* TLR2, TLR3 and TLR4 (Chang et al., 2017). Also, the M1 macrophages to produce MMP-10 contribute to vascular remodeling through STAT1 signaling (Chi et al., 2022). TGF-β secreted by CD4+ T cells is also involved in vascular reorganization (Li et al., 2020).

#### 2.3.3 Cancer-associated fibroblasts

Activated CAFs often build a matrix-dense barrier in the ECM to protect tumor cells and trap T cells, which affects the efficacy of ICIs (Godefroy et al., 2014). In metastatic urothelial carcinoma patients treated with PD-L1 monoclonal antibody, activated TGF-β signaling pathway in CAFs may hinder T cells from infiltrating tumors (Mariathasan et al., 2018). In co-culture with triple-negative breast cancer, TNF-α and IL-1β induced CXCL8/CCL5 secretion to suppress CD8+ T cells (Liubomirski et al., 2019). The resistance of breast cancer bone metastases to ICB is thought to be the release and resorption of TGF-β after osteoclast induction of osteogenesis, reducing the number of Th1 (Jiao et al., 2019). In addition, p-STAT-3, which is associated with metastatic brain tumors, reduces CD8+ T cell activity and increases the abundance of CD74+ macrophages, potentially playing a role in ICIs (Priego et al., 2018). CAFs have been reported to increase the expression of Fas and PD-1 to control the binding with PD-L2 for the former and Fas ligand for the latter (Lakins et al., 2018). Loss of Chitinase-3-like 1 in CAFs also increases the TILs (Cohen et al., 2017). Meanwhile, CAF inhibits NK protein (NKp) 44, 30, DNAX accessory molecule-1 (DNAM-1), and poliovirus receptors (Calon et al., 2014; TomokoInoueKatsuyukiTaguchi et al., 2016), which are the activating receptors of NK cells. High levels of IL-6 secreted by CAFs summon MDSCs and amplify PD-L1 in hepatocellular cancer (Liu et al., 2017). CSF-1 in the TME contributes to the reduction of chemotactic factors in CAFs. CSF-1 receptor and CXCR2 are key items for MDSCs gathering (Kumar et al., 2017b). CTLA-4 antibodies were more effective when combined with the GM-CSF vaccine (van Elsas et al., 1999). CAFs can also secrete other negative factors, such as CXCL12, CCL2, and VEGF (Grum-Schwensen et al., 2005; Chen et al., 2017). CXCL12/CXCL12R helps sensitize to anti-PD-L1 therapy, resulting in T cell aggregation and cancer regression (Feig et al., 2013). In gastric and colon cancers, fibroblast activation protein (FAP) in CAFs leads to more CCL2 and less IFN-γ, eventually damaging the outcome of ICIs therapy (Chen et al., 2017), while FAP Inhibition reverses this resistance (Wen et al., 2017). DNA vaccines against FAP induce activation of CD8+ and CD4+ T cells to enhance responses to ICIs (Xia et al., 2016). A pan-cancer analysis showed that TGF-β signaling in CAFs activated “ECM-up” signaling, independent of CAFs or TGF-β activation, a predictor associated with anti-PD-1 resistance, even better than T Cellular inflammatory signaling and mutational burden (Chakravarthy et al., 2018).

Given the complexity and dynamics of the tumor microenvironment, a single biomarker may not be sufficient, and a combination of multiple markers is supposed to be paneled (Binnewies et al., 2018; Kitano et al., 2018). Initial tumor biopsy assessment of PD-L1 abundance, TIL infiltration, and ECM status, combined with longitudinal assessment of changes in each factor during treatment, may have a unique potential to predict tumor phenotypes that respond to ICIs.

## 3 Inflammatory factors on immune-related adverse effects to immune checkpoint inhibitors in the circulation

Treatment with ICIs induces systemic and local inflammatory responses (Tsukamoto et al., 2018), starting from the interaction between inflammatory factors and their recruitment to immune cells. Inflammatory factors mediate each other. For example, the combination of anti-PD-L1 and anti-IL-6 antibodies increases IFN-γ and Th1 in melanoma mice (Tsukamoto et al., 2018), turning off IL-6/STAT3 pathway, which further activates IFN-γ/STAT1 for causing local inflammation (Xiang et al., 2020). In addition, inflammatory factors contribute to the accumulation of immune cells. TNF-α contributes to Th17 differentiation in colon cancer treated with anti-CTLA-4 (Beck et al., 2006), possibly due to IL-23/Th17 axis activation (Liu et al., 2021). In addition, eosinophils in the skin (Voskens et al., 2013) and macrophages in acute diabetes (Hu et al., 2020) are also the result of the application of ICIs by IFN-α or γ.

**FIGURE 3:**
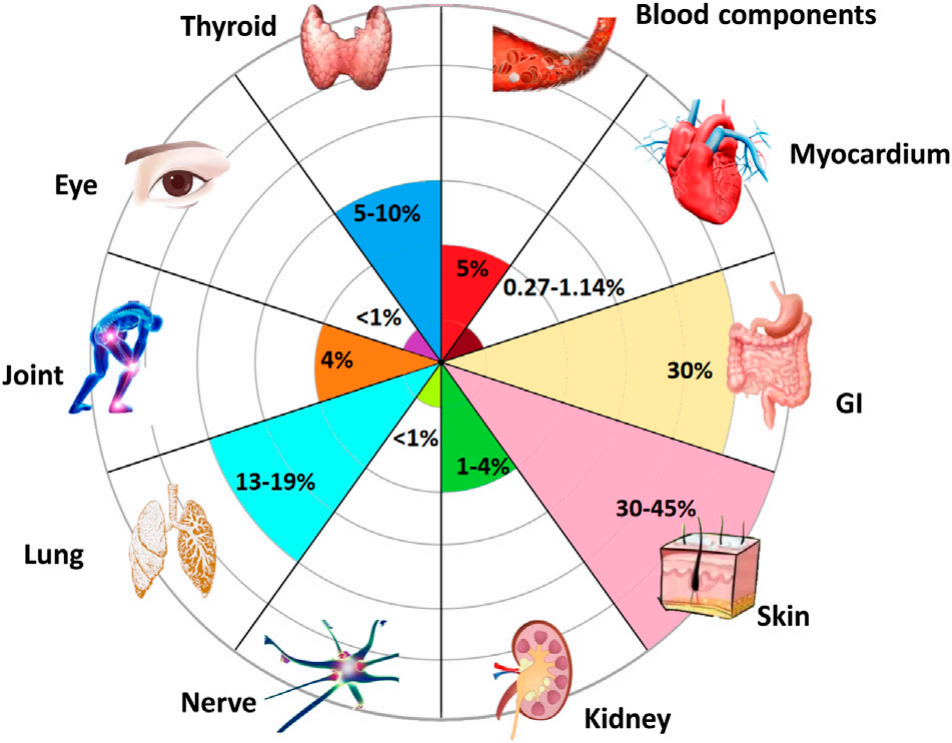
Organs susceptible to inflammatory toxicity induced by ICIs. Almost all organs may develop irAEs after receiving ICIs (Poto et al., 2022). Among them, the most common side effects occurred in skin, with an incidence rate of 40%. Gastrointestinal (GI) system with the occurrence of 30%. Followed by lungs and thyroid. The incidence of joints, blood components, and kidneys are all around 5%, while ocular, myocardial, and nerve involvement have been reported rare with a rate less than 1%.

Further, the expansion of the inflammatory factor cascade leads to immune damage to normal tissues, which is the main cause of side effects. IrAEs can occur in various organs (Figure 3) and are associated with good responses to ICIs (Saleh et al., 2019). However, an overresponse can hinder the course of ICIs treatment (Saleh et al., 2019), and the presence of severe irAEs leads to discontinuation of ICIs therapy temporarily or permanently according to guidelines. Induction of increased immune-promoting cells, such as a marked expansion of Th17 cells, is an independent risk factor for severe colitis in patients receiving ipilimumab (Tarhini et al., 2015). In contrast, immunosuppressive cells, such as Treg cells, tend to be depleted after treatment with ICIs (Simpson et al., 2013). The influx of myeloid cells also stimulates organ damage (Nam et al., 2019). In addition, different irAEs may have their specific cytokine profiles (Table 2). Circulating baseline IL-17 levels may help predict patients who are likely to develop severe diarrhea and colitis after treatment with ICIs (Young et al., 2018). However, IL-22 was not elevated (Luoma et al., 2020). The inflammatory response in rheumatoid arthritis is often accompanied by abundant TNF-α and IL-6 (Mcinnes et al., 2016). PD-1 blockade results in increases in IFN-γ, TNF-α, IL-2, 6, 17, and 23 (Dulos et al., 2012; Mcinnes et al., 2016). These changes may trigger psoriasis when treated with PD-1 inhibitors (Murata et al., 2017). However, IL-8 was not associated with dermatitis (Tanaka et al., 2017). In addition, upregulation of CXCL9, CXCL10, and CXCL11 has also been observed to correlate with PD-1 drug-related skin toxicity (Goldinger et al., 2016; Vivar et al., 2017). Prior to treatment, higher concentrations of IL-1β, 2, and GM-CSF were exposed in thyroid irAE (Kurimoto et al., 2020). Anti-TNF monoclonal antibodies prevent colitis (Perez-Ruiz et al., 2019), but at the same time may exacerbate cardiotoxicity associated with ICIs (Kwon et al., 2003) and may exacerbate interstitial lung disease (Akiyama et al., 2016). Pneumonitis, serum sickness, encephalitis, and systemic inflammatory response syndrome were all improved when anti-IL-6 antibodies were used to treat severe IRAE (Stroud et al., 2019). However, it increases the risk of inflammatory bowel disease (Korzenik et al., 2019). Arthritis induced by ICIs can also be ameliorated by inhibition of IL-6 and TNF-α (Sang et al., 2017; Cappelli et al., 2018). Other cytokines, such as IL-12p70, 1α, 1RA and 13, IFN-α2, CSF, CX3CL1, and FGF-2, were also differentially expressed in the plasma of severe irAEs patients before and during ICIs treatment (Tarhini et al., 2015), but they The association with specific classes of IRAEs and their associated mechanisms are unclear.

**TABLE 2:** Differences of irAEs in Inflammatory Factors.

	irAEs	Inflammatory factors	References	Anti-TNF	References	Anti-IL6	References
Digestive system	Diarrhea, colitis	IL-17	Young et al. (2018)	Efficient	Perez-Ruiz et al. (2019)	Aggravate	Korzenik et al. (2019)
Endocrine System	Thyroiditis	IL-1β, 2, GM-CSF	Kurimoto et al. (2020)	—		—	
Circulatory system	Myocarditis	—		Aggravate	Kwon et al. (2003)	—	
Motor system	Arthritis	TNF-α, IL-6	Mcinnes et al. (2016)	Efficient	Cappelli et al. (2018)	Efficient	Sang et al. (2017)
Respiratory system	Pneumonia	—		Aggravate	Akiyama et al. (2016)	Efficient	Stroud et al. (2019)
Nervous system	Demyelination, encephalitis	—		—		Efficient	Stroud et al. (2019)
Skin	Psoriasis, alopecia areata	IL-2,6,17,23,CXCL9,10,11, IFN-γ, TNF-α	Dulos et al. (2012), Mcinnes et al. (2016), Murata et al. (2017), Goldinger et al. (2016), Vivar et al. (2017)	—		—	

## 4 Conclusion and future perspectives

Less than half of the cases respond to ICIs (Young et al., 2018), and there is a lack of effective predictors. A personalized panel composed of various inflammatory factors is a proposed predictive tool, the main factors including IFN, TLR, IL, TNF-α and TGF-β. They were selected for affecting PD-L1 expression and immune cell infiltration. They not only activate signaling pathways or regulate chemokines but also participate in ECM remodeling or balance pro- and anti-inflammatory effects.

IrAEs occurred more frequently when ICIs treatment was shifted from monotherapy to combination therapy (Johnson et al., 2020). Balancing anti-inflammatory and pro-inflammatory is expected to resolve the dilemma between anticancer and anti-irAEs of ICIs therapy, so some combination drugs have been tried other than glucocorticoids. The first is inflammatory pathway inhibitors. PI3K blockade fights cancer and reduces side effects by silencing inflammatory pathways (Eschweiler et al., 2022). The second is inflammatory factor inhibitors, which have a bidirectional effect. Anti- and pro-inflammatory are regulated by TNF through binding to different receptors (TNFR1 and TNFR2) (Chen et al., 2021), and by IL-6 through activation of different signals (JAK-STAT3 or PI3K-Akt) (Nguyen et al., 2014). The third is small molecule inhibitors of immune checkpoints and nanoparticles (Zhang et al., 2019). Small-molecule inhibitors can reduce affinity to reduce IRAEs, while nanoparticles facilitate targeting to improve anticancer efficacy.

The influence of inflammatory factors is not only applicable to ICIs treatment, but also to other immunotherapies. Immune-related side effects are triggered consistently across various immunotherapies, all resulting from cytokine cascades (Weber et al., 2015). For example, in adaptive immune cell therapy, IL-6, IL-1 are the main triggers of cytokine release syndrome. Likewise, up-regulated IFN and IL-2 caused gastrointestinal reactions and thyroid damage during cytokine therapy. Due to the universality of the effects and side effects of inflammatory factors in various immunotherapies, the understanding of inflammatory factors in ICIs may be extended to immunotherapy in pan-cancer and benefit more patients.
